# Supplementary material for: Multi-epitope vaccine design using in silico analysis of glycoprotein and nucleocapsid of NIPAH virus
Source: PLoS One. 2024 May 10;19(5):e0300507. doi: 10.1371/journal.pone.0300507 (PMC11086869; doi:10.1371/journal.pone.0300507)
Supplement: Attachment — Submitted filename: comment.docx [file pone.0300507.s002.docx]

Dear authors please consider the mentioned comments related to manuscript entitled “Multi-Epitope Vaccine Design Using in silico analysis of Glycoprotein and Nucleocapsid of NIPAH virus”

1. Please mention in the manuscript the threshold, accuracy and URL for all used servers in epitopes selection and vaccine design. Also the references for all used servers should be mentioned in manuscript.
2. The conformational B cell epitopes should be selected.
3. This is a bioinformatics study, so it is better to change the section of materials and methods to methods.
4. Please, the authors answer to this question, what is the reason for doing docking with different TLRs (3, 7, and 8)?
5. It is suggested to refer to the following paper in the use section of the EAAAK, GPGPG and kk linkers.

<https://pubmed.ncbi.nlm.nih.gov/37146081/>

6. The references should be updated.
